# Association of microRNAs with Types of Leaf Curvature in *Brassica rapa*

**DOI:** 10.3389/fpls.2018.00073

**Published:** 2018-02-06

**Authors:** Wenqing Ren, Han Wang, Jinjuan Bai, Feijie Wu, Yuke He

**Affiliations:** ^1^National Key Laboratory of Plant Molecular Genetics, CAS Center for Excellence in Molecular Plant Sciences, Shanghai Institute of Plant Physiology and Ecology, Shanghai Institutes for Biological Sciences, Chinese Academy of Sciences, Shanghai, China; ^2^Jiangsu Key Laboratory for Biofunctional Molecules, College of Life Science and Chemistry, Jiangsu Second Normal University, Nanjing, China

**Keywords:** *Brassica rapa*, heading Chinese cabbage, leaf curvature, miR165/6, miRNA, transgenic plant

## Abstract

Many vegetable crops of *Brassica rapa* are characterized by their typical types of leaf curvature. Leaf curvature in the right direction and to the proper degree is important for the yield and quality of green vegetable products, when cultivated under stress conditions. Recent research has unveiled some of the roles of miRNAs in *Brassica* crops such as how they regulate the timing of leafy head initiation and shape of the leafy head. However, the molecular mechanism underlying the variability in leaf curvature in *B. rapa* remains unclear. We tested the hypothesis that the leaf curvature of *B. rapa* is affected by miRNA levels. On the basis of leaf phenotyping, 56 *B. rapa* accessions were classified into five leaf curvature types, some of which were comparable to miRNA mutants of *Arabidopsis thaliana* in phenotype. Higher levels of miR166 and miR319a expression were associated with downward curvature and wavy margins, respectively. Overexpression of the *Brp*-*MIR166g-1* gene caused rosette leaves to change from flat to downward curving and folding leaves to change from upward curving to flat, leading to the decrease in the number of incurved leaves and size of the leafy head. Our results reveal that miRNAs affect the types of leaf curvature in *B. rapa*. These findings provide insight into the relationship between miRNAs and variation in leaf curvature.

## Introduction

The leaf is the main site of photosynthesis, which produces sugars from water and carbon dioxide under sunlight. Additionally, leaves can be adapted for other purposes, including human consumption. The paleohexaploid *Brassica rapa* includes various crops that show very different leaf morphologies (Xiao et al., 2014). This species includes more than 13 vegetable crops that produce edible products such as leafy heads, curved leaves, modified blades, and modified petioles.

Heading Chinese cabbage (*B. rapa* ssp. *pekinensis*), non-heading Chinese cabbage (*B. rapa* ssp. *chinensis*) and turnip (*B. rapa* ssp. *rapifera*) belong to *B. rapa* (Li, 1990). Heading Chinese cabbages have leafy heads composed of extremely inwardly curved blades on their shoot tips, the leaves of non-heading Chinese cabbage show clusters of incurved petioles and outward folding blades and turnip displays flat leaves with a fleshy root. The distinct morphologies exhibited by subspecies of *B. rapa* represent some of the most spectacular evolutionary changes and illustrate the structural evolution of plants under domestication. The leafy head, the edible part of heading Chinese cabbage (*B. rapa* ssp. *pekinensis*), is composed of numerous heading leaves that usually curve after the rosette stage. Heading Chinese cabbage plants go through four vegetative phases during their vegetative growth period: the seedling, rosette, folding and heading stages. The rosette leaves differentiate at the rosette stage and function as photosynthetic organs, while the heading leaves are incurved to form heads and thus become storage organs for essential nutrients. Leafy heads have many different shapes, even though all their internal leaves curve inward. The leaves of upright heads are erect and curved transversely, while those of heads with a flat top are curved transversely and longitudinally, and overlap at the upper parts. The leaves of heads with a joined top are curved transversely and longitudinally, but without overlapping at the upper parts. The production of Chinese cabbage is often affected by poor heading. Temperature, light intensity, and photoperiod all affect the formation of leafy heads (Ito and Kato, 1957, 1958). Additionally, endogenous auxin can modify the processes of leaf bending and folding (He et al., 1994, 2000).

For non-heading Chinese cabbage (*B. rapa* ssp. *chinensis*), the petioles are curved upward or downward. They are not able to form leafy heads, but develop into curved leaves full of vitamins, minerals and nutrients. For some crops, the petioles are thickened and curved, forming fleshy petioles. The quality of edible leaves is largely dependent on leaf (include petiole) shape, size, angle, color, and curvature.

Almost all crops of *B. rapa* are characterized by their typical leaf curvature. Interestingly, most cultivars differ in the direction, axis, position and/or degree of leaf curvature. For a long time, the quantification of leaf curvature was not possible, and thus comparisons of leaf curvature between species and/or between crops has been difficult. However, Liu et al. (2010) proposed a formula to quantify the degree of leaf curvature of *Arabidopsis* mutants deficient in miRNA pathways. We wondered whether this formula was suitable for the quantification and characterization of all types of leaf curvature in *B. rapa*. Moreover, it remains unknown whether various types of miRNAs are involved in the determination of these types of leaf curvature. Thus, we also wondered how minimal morphological changes of leaf curvature were related to miRNAs or miRNA-targeted genes.

In *Arabidopsis*, many miRNAs are involved in leaf flatness (Liu et al., 2011). miRNA accumulation is controlled by DICER-LIKE1 (DCL1), HYPONASTIC LEAVES1 (HYL1), SERRATE (SE), and AGONAUTE1 (AGO1) (Park et al., 2002; Reinhart et al., 2002; Han et al., 2004; Vaucheret et al., 2004; Wu et al., 2007). Nearly all non-lethal mutants of these genes show morphological, physiological, and biochemical changes. Mutant plants deficient in polarity, cell division and the auxin response exhibit abnormal leaf curvature. miR165/166 targets five members of the class III homeodomain leucine zipper (HD-ZIP III) family (McConnell and Barton, 1998; Sessa et al., 1998; Emery et al., 2003; Nath et al., 2003; Byrne, 2006). The dominant mutation of MIR166g in *Arabidopsis* causes downward curvature of leaves (Williams et al., 2005). miR319a targets the *TEOSINTE BRANCHED1*/*CYCLOIDIA*/*PCF* (*TCP*) gene family (Palatnik et al., 2003). Ectopic expression of miR319/JAW causes a wavy-leaf phenotype (Palatnik et al., 2003). TAS3 ta-siRNA degrades *ARF3* and *ARF4* mRNAs (Peragine et al., 2004; Allen et al., 2005; Xie et al., 2005; Fahlgren et al., 2006). miR164 targets *CUC1* and *CUC2* (Ernst et al., 2004; Olsen et al., 2004), which are necessary for the formation of boundaries between meristems and emerging organ primordia (Aida et al., 1999; Mallory et al., 2004; Nikovics et al., 2006). Using miR164ts-tagged KRP1, growth repression in the distal region of the leaf was shown to lead to goose foot-shaped leaves (Malinowski et al., 2011). The divergence in leaf curvature in higher plants has already been described. However, few developmental genetic and evo/devo studies have been carried out, especially in crops (Tsukaya, 2014).

Interestingly, the leaf curvature in many *B. rapa* crop resembles that of mutants or transgenic plants of *Arabidopsis* miRNA genes or miRNA target genes. In Chinese cabbage, overexpression of the *MIR319a* gene silences miR319a-targeted *TCP* genes, causing extra cell division in the marginal regions of leaves that results in wavy margins, and bulging and straightening of the top regions in heading leaves (Mao et al., 2014). Overexpression of *BrpSPL9-2* caused significant premature leaf incurvature and heading while overexpression of miR156 delayed leaf curvature (Wang et al., 2014). We propose that different types of leaf curvature in *B. rapa* are modified by miRNAs. In this report, we tested this hypothesis by examining the association between miRNA levels and leaf curvature. Our results provide insight into the relationship between miRNAs and variations in leaf curvature.

## Materials and methods

### Plant materials

The three crop types used in this study are heading Chinese cabbage (*B. rapa* ssp. *pekinensis*), non-heading Chinese cabbage (*B. rapa* ssp. *chinensis*), and turnip (*B. rapa* ssp. *rapifera*). Heading Chinese cabbage include the crops: Sanye (ssp. *pekinensis* var. *dissoluta* Li), Banjieqiu (ssp. *pekinensis* var. *infarcta* Li), Huaxin (ssp. *pekinensis* var. *laxa* Tsen et Lee) and Jieqiu (ssp. *pekinensis* var. *cephalata* Tsen et Lee) while non-heading Chinese cabbage include the crops: Baicai (ssp. *chinensis* var. *communis*), Wutacai (ssp. *chinensis* var. *rosularis*), Caitai (ssp. *chinensis* var. *utilis*), Zicaitai (ssp. *chinensis* var. *purpurea*) and Taicai (ssp. *chinensis* var. *taitsai*) (Li, 1990). Each crop type consists of several genotypes with different types of leaf curvature. In total, 56 genotypes were used for characterization of leaf curvature. All of them are the inbred lines except for two turnip cultivars. The genotypes used in this study are presented in Supplemental Table S1.

For comparison with the genotypes of Chinese cabbage, *Arabidopsis* mutants deficient in miRNA-mediated pathways were also selected.

The seeds of Chinese cabbage and *Arabidopsis* were sown in petri dishes with moistened filter paper and sealed with parafilm, moved to a growth chamber and grown at 22°C with 16 h of light. Four days later, they were transplanted to a growth chamber in the SIPPE phytotron and transferred to the field at the experimental station of SIPPE, Songjiang, Shanghai. More than 20 individual plants for each crop were sampled for various measurements.

### Quantitative measurement of leaf curvature

The leaves were labeled at different developmental stages for quantitative measurement of leaf curvature. Before leaf flattening, we defined the type and direction of leaf curvature. To measure the global transverse curvature, we fixed two points, *a* and *b*, on the two lateral margins of the leaf at the widest point (Liu et al., 2011). Curvature indexes (CIs) for downward and upward curvature were calculated using the formulae *CI* = (*a*′*b*′ − *ab*)/*a*′*b*′ and *CI* = (*ab* − *a*′*b*′)/*a*′*b*′, respectively, where *ab* is the distance between points *a* an *b* on the two margins before leaf flattening and *a*′*b*′ is the distance between *a* and *b* on the two margins after flattening. The number of leaves for each measurement was more than 20. The significant differences were referred to *P* < 0.001 (Student's *t*-test) between genotypes.

### RNA sampling and miRNA microarray analysis

The leaf samples were harvested from the 3-week-old seedlings of different genotypes and frozen immediately in liquid nitrogen. Then, the materials were sent to ShanghaiBio Corporation where the RNAs were isolated for miRNA microarray. Isolation of total RNA is done by the phenolic separation of DNA and RNA using Trizol (Invitrogen, Carlsbad, CA). The purification of miRNA samples, labeling, and hybridization analysis were performed following the instructions (Liu et al., 2008). Each sample was assayed in duplicate. The data were extracted using LuxScan (CapitalBio), and the differential miRNAs were selected using SAM (Significance Analysis of Microarrays ver. 3.0).

### Real-time PCR

Total RNA was extracted from seedlings using TRIzol (Invitrogen) and treated with DNase I (TaKaRa) to remove DNA contamination. Four micrograms of RNA was used for reverse transcription with oligo(dT) primers. PCR was performed with the Rotor-Gene 3000 system (Corbett Research) using SYBR Premix Ex Taq (TaKaRa) according to the manufacturer's instructions. *BrpACTIN* mRNA was used as an internal control. The comparative threshold cycle (Ct) method was employed to determine relative transcript levels (MyiQ2 two-color real-time PCR detection system; Bio-Rad). Expression was normalized relative to that of *Brp*ACTIN. Three biological replicates and three technical replicates were performed. The significant differences were referred to *P* < 0.001 (Student's *t*-test) between genotypes.

### Genetic transformation

The *Brp-MIR166g-1* gene was cloned by PCR from seedlings of heading Chinese cabbage (Bre) and inserted into pCAMBIA1300 binary vectors under the control of the AA6 promoter. The binary constructs were delivered into *Agrobacterium tumefaciens* strain GV3101(pMP90RK) (Weigel and Glazebrook, 2006). The Bre plants were transformed using a vernalization-infiltration method as described previously (Bai et al., 2013). The transgenic plants of the T1 generation were pollinated, and homozygotes were selected from the T2 and T3 populations.

## Results

### Variation of leaf curvature in *B. rapa*

**Figure 1:**
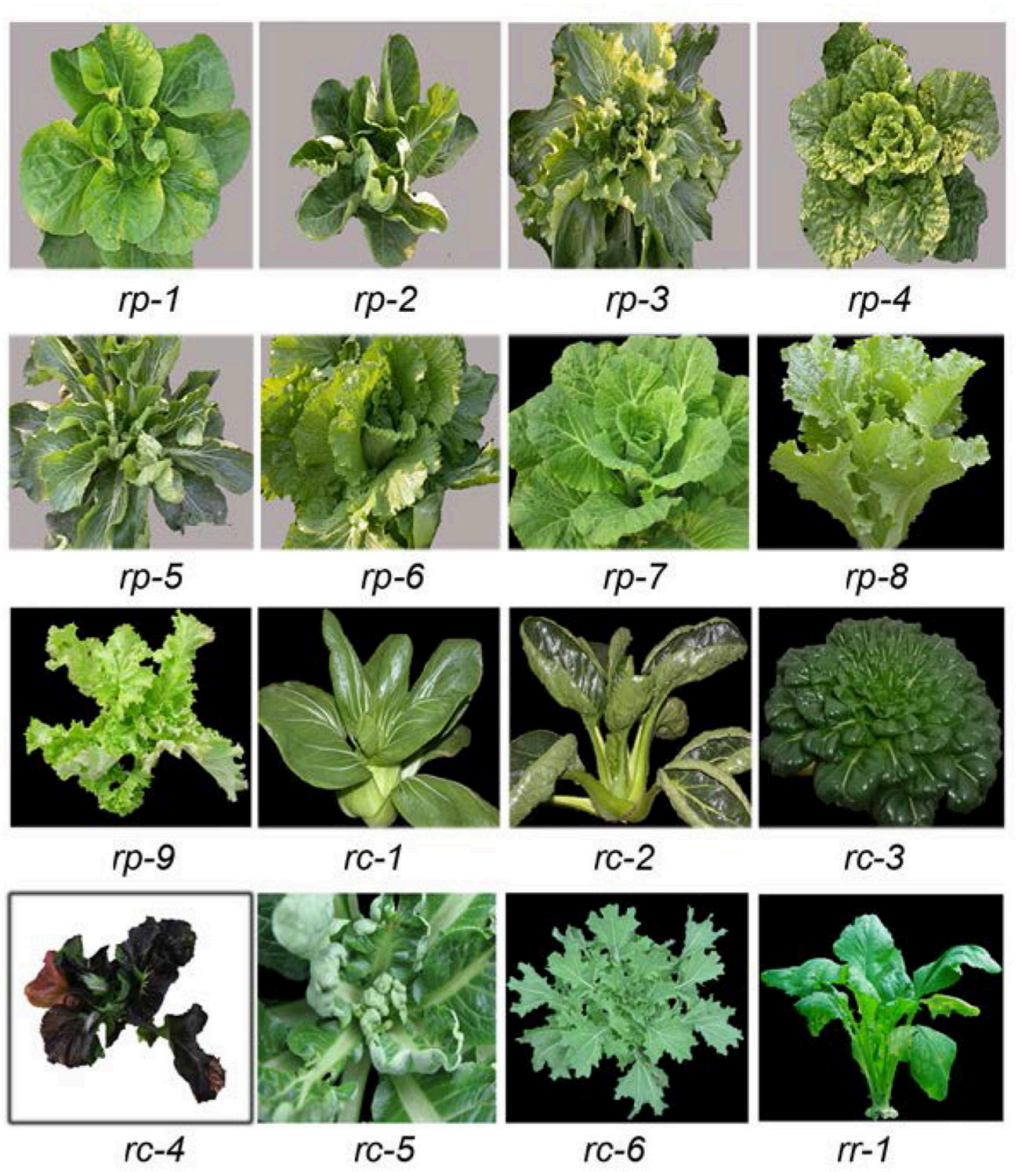
Diversity of leaf curvature in *B. rapa*. *rp-1* through *rp-9* are nine genotypes of heading Chinese cabbage (*B. rapa* ssp. *pekinensis*) with different types of leaf curvature. *rc-1* through *rc-6* are six genotypes of non-heading Chinese cabbage (*B. rapa* ssp. *chinensis*) with different types of leaf curvature. *rr-1* is a genotype of turnip (*B. rapa* ssp. *rapifera*).

*B. rapa* shows a broad spectrum of leaf curvature and morphological variation. To characterize the direction, degree and position of leaf curvature in *B. rapa*, we selected the 14th leaves (rosette leaves) from a collection of 56 accessions for observation and measurement (Supplemental Table S1). The accessions of heading (*rapa* ssp. *pekinensis*), non-heading (*rapa* ssp. *chinensis*), and turnip (*rapa* ssp. *rapa*) were named with the initials *rp, rc*, and *rr*, respectively. Most accessions showed global curvature in the transverse or longitudinal axis of the leaves, as the entire leaves curved downward or upward (Figure 1). However, some accessions displayed local leaf curvature as local regions of the leaf such as the blade tip, margin and petiole displayed certain types of curvature.

During plant growth, the direction, degree, and position of leaf curvature and the inclination angle are changed at different development stages. For convenience of comparison, we chose the 14th leaf of plants for observation of leaf curvature. Among 56 accessions, 16 representative genotypes with different leaf curvature were given in Table 1 and Figure 1.

**Table 1:** Different types of leaf curvature in *B. rapa*.

**Crop types**	**Curvature types**	**Phenotypes**	**Genotypes**
Heading Chinese cabbage	Flat	Flat leaves	*rp-1*
		Flat leaves, shrunk	*rp-4*
	Downward curving leaves	Leaves downward curving in longitudinal direction	*rp-3, rp-5, rp-9*
	Serrated blades	Serrated blades, blades downward curving in longitudinal direction	*rp-3*
		Serrated blades, blades upward curving in transverse and longitudinal directions	*rp-6*
		Serrated blades, blades upward curving in transverse direction and downward curving in longitudinal directions	*rp-8*
	Wavy margins	Wavy margins, blades upward curving in transverse direction and downward curved in longitudinal direction	*rp-3,rp-5, rp-9*
		Wavy margins, blades upward curving in transverse and longitudinal directions	*rp-6, rp-8*
	Upward curving leaves	Leaves upward curving in transverse and longitudinal directions	*rp-2, rp-6, rp-7, rp-8*
		Leaves upward curving in transverse and downward curving in longitudinal directions, wavy margins	*rp-3, rp-5, rp-9*
Non-heading Chinese cabbage	Flat	Flat blades	*rc-1*
		Flat blades, deeply serrated	*rc-6*
	Downward curving leaves	Leaves downward curving	*rc-3, rc-4*
	Inward curving petioles	Petioles inward curving	*rc-2, rc-5*
	Serrated blades,	Blades upwardly curving in transverse and longitudinal directions	*rc-4*
	wavy margins	Wavy margins	*rc-4*
		Wavy margins, bulged	*rc-5*
	upward curving leaves	Blades upward curving in transverse and longitudinal directions, bulged	*rc-2*
		Blades upward curving in transverse and longitudinal directions, bulged, wavy margins	*rc-5*
Turnip	Downward curving leaves	Blades downward curving, lobed	*rr-1*

*The 14th leaves of all genotypes were observed and classified according to the direction, degree, and position of leaf curvature*.

According to the direction, degree and position of curvature and the inclination angle, we classified the 56 accessions into five types of leaf curvature: downward curving leaves, inward curving petioles, serrated blades, wavy margins, and upward curving leaves. Upward curving leaves (rosette leaves along both the transverse and longitudinal axes) were observed in *rp-2, rc-2*, and *rc-5* plants; upward curving petioles (small inclination angle of petioles) in *rc-3* plants; serrated blades in *rc-6* plants; wavy margins in *rp-6* plants; and downward curving leaves (rosette leaves along both the transverse and longitudinal axes) in *rr-1* plants.

In many cases, global curvature was mixed with local curvature in a single leaf and wavy margins, deep serration, upward curvature, and inward curvature appeared together. For example, *rc-3* leaves curved downward as a whole; their apical regions curved downward, but their central regions curved upward. In such cases, global and local curvature were measured separately. Accordingly, some genotypes were categorized into two or three types of leaf curvature.

### Leafy head shapes

**Figure 2:**
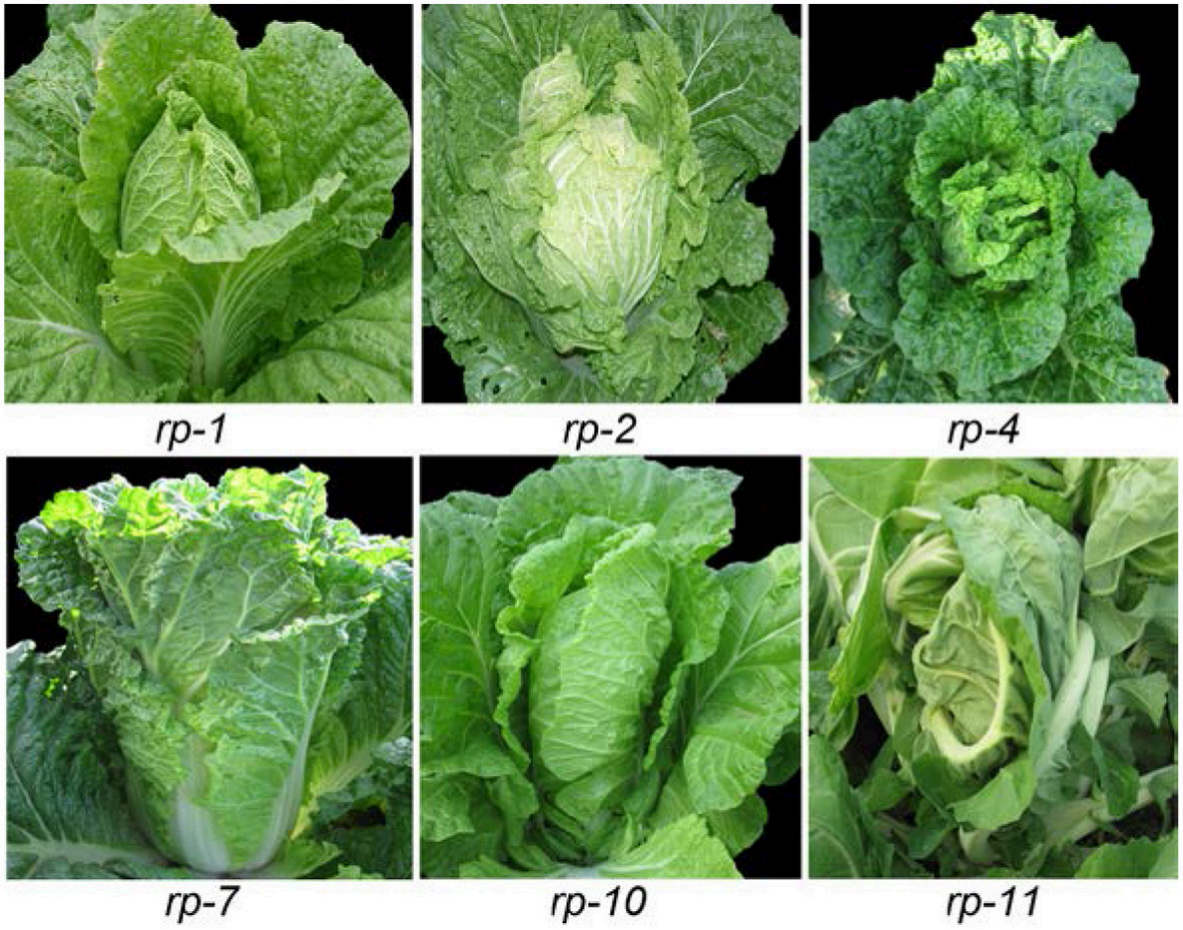
Leafy head shape of heading Chinese cabbage. *rp-1, rp-2, rp-4, rp-7, rp-10*, and *rp-11* are six genotypes of heading Chinese cabbage.

**Figure 3:**
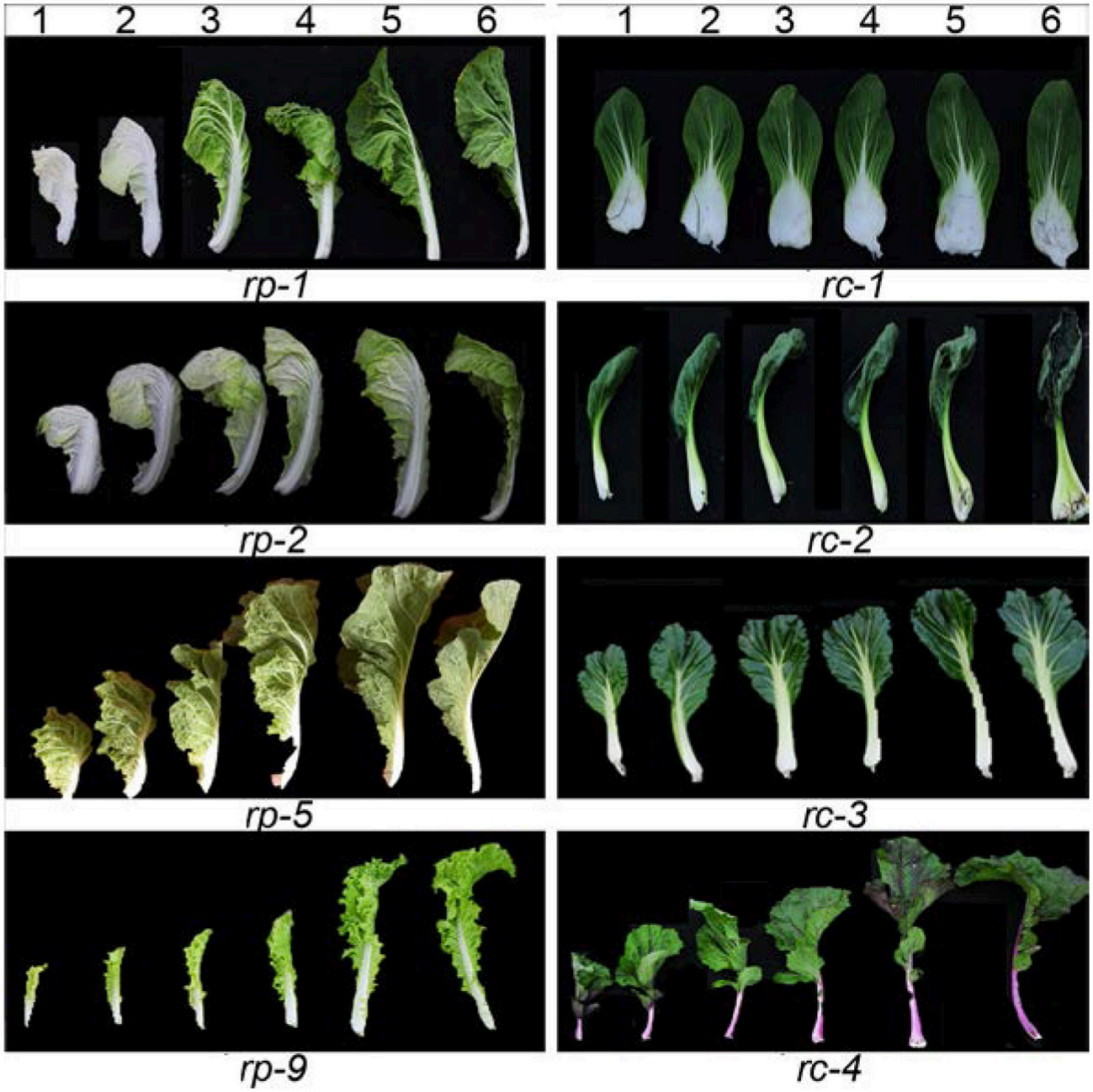
Direction and degree of leaf curvature in different leaves. *rp-1, rp-2, rp-5*, and *rp-9* are the four genotypes of heading Chinese cabbage, while *rc-1, rc-2, rc-3*, and *rc-4* are the four genotypes of non-heading Chinese cabbage. Leaf 6 at the far right is the first leaf of the edible parts while leaf 1 at the far left is the inner leaf.

Heading Chinese cabbage shows different head shapes. The head tops of heading Chinese cabbage vary due to the diversity of leaf curvature. As shown in Figure 2, the *rp-1* heads were oval with a cone-shaped top, as the leaf tips curved upwardly. The *rp-2* heads were round as the leaf tips curved inwardly and thus overlapped. The *rp-7* heads were cylindrical as the leaf incline angle became small or near zero. The *rp-10* heads were upright with overturned leaf tips, due to downward curvature of the leaf tips. The *rp-11* heads were elliptical as the head leaves were twisted in a counter-clockwise direction. Inside the leafy heads of heading Chinese cabbage, the head leaves were extremely incurved compared with those in the rosette. To compare the phenotypes of the different head leaves among crops, we harvested the leaves of leafy heads progressively from the outside to the inside and observed the direction and degree of leaf curvature in order. As shown in Figure 3, the leaves in different types of leafy heads were distinct in the direction and degree of curvature. For *rp-1* leaves, the outermost leaf (leaf 6) of the leafy head curved inward transversely while the inner leaf (leaf 1) curved inward longitudinally and transversely. For *rp-2* leaves, both the outermost and innermost leaves curved inward along both the longitudinal and transverse axes, and the degree of leaf curvature increased progressively from leaf 6 to leaf 1. For *rp-5* leaves, the outermost leaves of the leafy head curved inward transversely but outward longitudinally while the inner leaves curved inward only transversely. For *rp-9* leaves, both the outermost and innermost leaves curved inward transversely but outward longitudinally, even though all the leaves had wavy margins.

### Incline angles of the leaf, blade, and petiole

**Figure 4:**
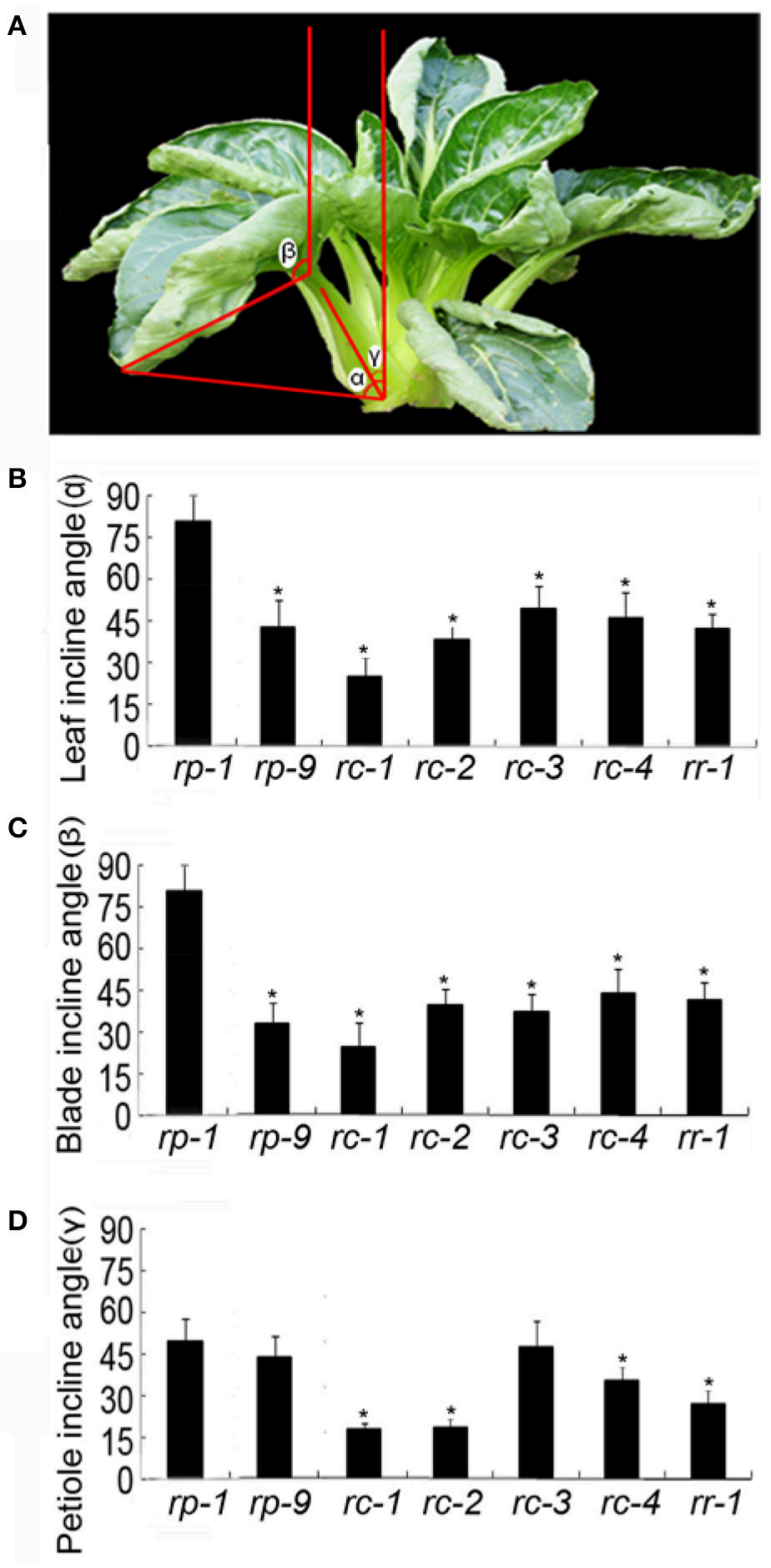
Incline angles of the leaf (α), blade (β), and petiole (γ) of different genotypes. **(A)** *rc-2* plant. **(B–D)** Incline angles of the leaf (α) **(B)**, blade (β) **(C)**, and petiole (γ) **(D)**. *rp-1* and *rp-9* are two genotypes of heading Chinese cabbage, *rc-1, rc-2, rc-3*, and *rc-4* are four genotypes of non-heading Chinese cabbage, and *rr-1* is one genotype of turnip. Error bars represent the standard deviation (*SD*). The number of leaves measured for each genotype was >20. The significant differences were referred to *P* < 0.001 (Student's *t*-test) between genotypes. ^*^Indicates the significant difference.

The shapes of the leaves and leafy heads of *B. rapa* plants are not only related to the curvature of leaves, but also to leaf incline angles. All leaves in *B. rapa* plants are inclined at various angles to the vertical axis from 0° to 180°. In Figures 4A,B, the leaf angle, blade angle and petiole angle of a *rc-2* plant represented by α, β, and γ, respectively. If α = 0, the leaf was upright, and if α = 90°, the leaf was flat to the horizontal plane. Interestingly, the leaf angle, blade angle and petiole angle of this plant were distinct. The blade angle β was more than 90° while the leaf angle α and petiole angle γ were less than 90°. From the outer to the inner leaves, the leaf angle, blade angle and petiole angle became progressively smaller. We noticed that the petiole angles of *rc-1* plants were so small that the petioles were bundled into a cluster.

Leaf incline angles varied by accessions and not by the crop types. At the rosette stage, the leaf angles were ranked *rp-1*>*rc-3*>*rc-4*>*rp-9*>*rr-1*>*rc-2*>*rc-1* (Figure 4). The petiole angle was greatest in *rp-1* and smallest in *rc-1*. The leafy head of *rp-7* was special in that the leaf, blade and petiole angles were almost the same and were all nearly zero, meaning that the leaves were erect. We noticed that the outer leaves around the *rp-7* head remained inclined at various angles, distinct from the internal leaves in the leafy head. Importantly, a small leaf incline angle or leaf erectness is necessary for the formation of leaf heads.

### Curvature indices of leaves

**Figure 5:**
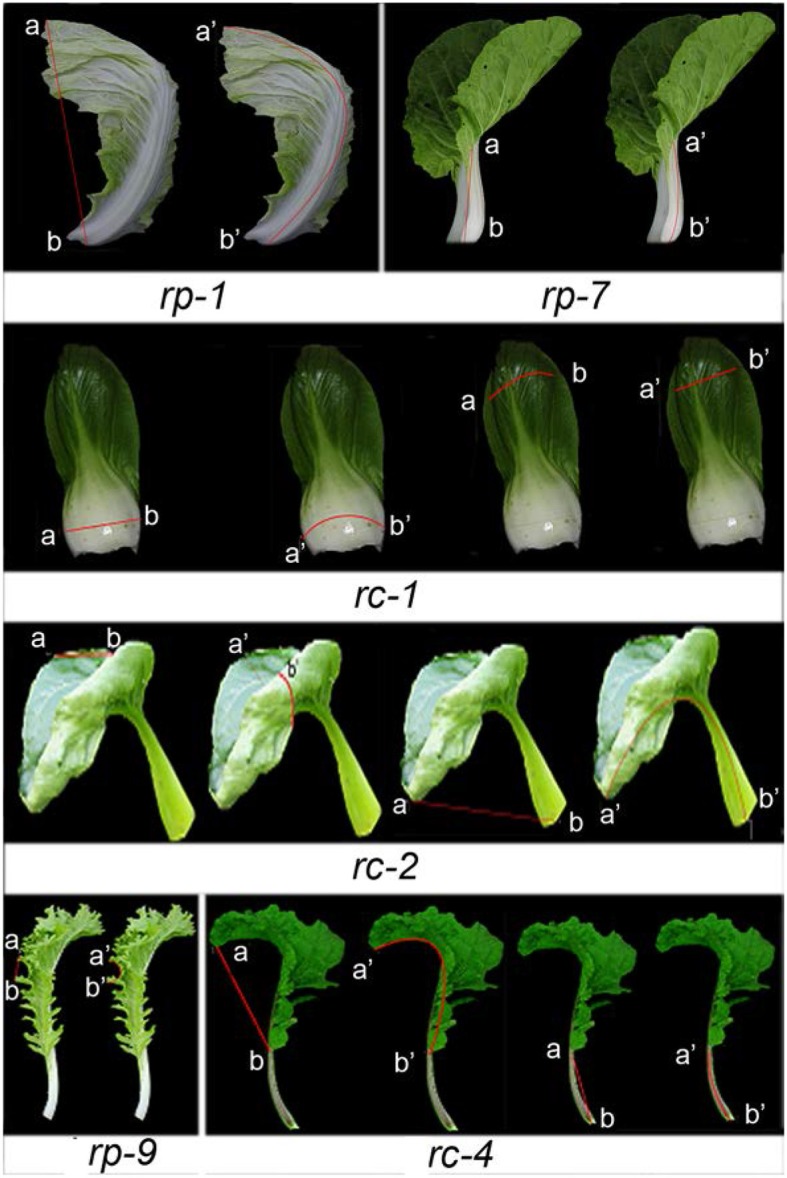
Measurement of leaf curvature. *rp-1, rp-7*, and *rp-9* are three genotypes of heading Chinese cabbage, *rc-1, rc-2*, and *rc-4* are three genotypes of non-heading Chinese cabbage. The straight distance between *a* and *b* was designated as the projected width, while the distance between *a*′ and *b*′ was designated as the flattened width.

**Figure 6:**
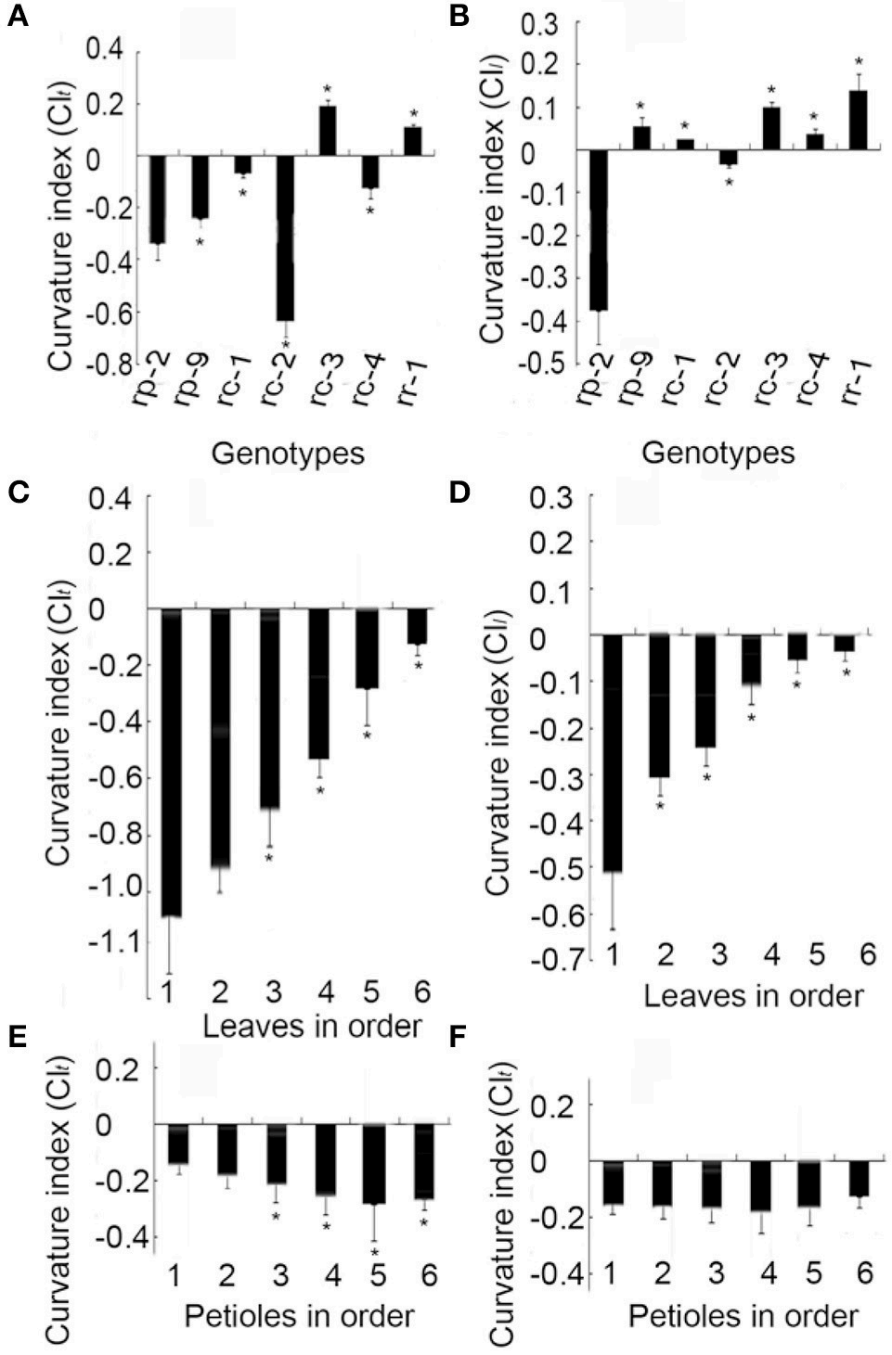
Transverse and longitudinal curvature indices (CIs) of leaves and petioles. *rp-2* and *rp-9* are two genotypes of heading Chinese cabbage, *rc-1, rc-2, rc-3*, and *rc-4* are four genotypes of non-heading Chinese cabbage, and *rr-1* is one genotype of turnip. **(A,B)** CIs of leaves in different genotypes. **(C,D)** Transverse and longitudinal CIs of the inner to outer leaves of leafy heads of *rp-2*. 1–6 on the abscissa axis indicate six leaves with 1 as the innermost leaf and 6 as the outermost leaf. **(E,F)** Transverse and longitudinal CIs of petioles from the outer to inner leaf clusters in *rc-1*. 1–6 on the abscissa axis indicate six leaves with 1 as the innermost leaf and 6 as the outermost leaf. Error bars represent the standard deviation of more than 20 seedlings. The significant differences were referred to *P* < 0.001 (Student's *t*-test) between genotypes. ^*^Indicates the significant difference.

In *Arabidopsis*, leaf curvature was quantified using the formula CI = (*a*′*b*′ – *ab*)/*a*′*b*′, where *ab* is the distance between points *a* and *b* on the two margins of a leaf before flattening, and *a*′*b*′ is the distance between *a* and *b* on the two margins after flattening (Liu et al., 2010). To determine whether this formula was applicable to *B. rapa*, we calculated CI values for several representative types of leaf curvature (Figure 5). In *rp-1* heading leaf, the straight-line distance between *a* and *b* along longitudinal axis before flattening is shorter than the one between *a*′ and *b*′ after flattening. In *rc-1* petiole, the straight-line distance between *a* and *b* along transverse axis before flattening is shorter than the one between *a*′ and *b*′ after flattening. *rc-2*, a genotype of non-heading Chinese cabbage, had the lowest (−0.63) transverse CI while *rc-3*, another genotype of non-heading Chinese cabbage, had the highest (0.19) transverse CI for blades (Figure 6A). *rp-2*, a genotype of heading Chinese cabbage, had the lowest (−0.37) longitudinal CI for blades while *rr-1*, a genotype of turnip, had the highest (0.14) (Figure 6B). Although the leaves of these genotypes showed global curvatures, the directions of leaf curvature along the transverse and longitudinal axes were different.

The CIs of leaves were related to the order of the leaves on the shortened stem. In *rp-2* plants, either the transverse or longitudinal CI increased progressively from the innermost to outermost leaves in the leafy head (Figures 6C,D), in which the transverse and longitudinal CIs of the innermost blades were −1.03 and −0.51 while those of the outermost blades were −0.11 and −0.03. In *rc-1* plants, both the transverse and longitudinal CIs of the petioles decreased progressively from leaves 1 to 4 (Figures 6E,F), indicating that the severity of petiole inward curvature increased from the outer to inner leaves. Specifically, the transverse and longitudinal CIs of the innermost petioles were −0.15 and −0.16 while those of the outermost blades were −0.24 and −0.18.

### Comparison of leaf curvature in *B. rapa* with that of arabidopsis

The various types of leaf curvature in different genotypes of *B. rapa* were similar to many mutants of *Arabidopsis* deficient in miRNA-directed pathways. In total, 22 *Arabidopsis* lines including mutants deficient in miRNA biogenesis, *MIRNA* genes and miRNA-targeted genes, and transgenic lines related to leaf curvature were selected. To examine the possible relationship between miRNAs and leaf curvature in *B. rapa*, we compared the leaf shapes of the different genotypes with those of previously reported miRNA-deficient *Arabidopsis* mutants. The downward curvature of *rc-3* leaves resembled that of the *jba-1D* mutant in which the *MIR166g* gene is activated (Williams et al., 2005). The wavy margins of *rp-3, rp-9, rc-4*, and *rc-5* leaves (Figure 1) were similar to those of the *jaw-1D* mutant in which the *MIR319a* gene is activated (Palatnik et al., 2003; Table 1). The upward curvature of *rp-2, rc-2*, and *rc-5* plants resembled that of *35S::mCNA* plants in which the miR165/6-targeted *CNA* gene is overexpressed (Liu et al., 2010), and the downward curvature of *rc-3* leaves looked like that of the *rev-6* mutant (Prigge et al., 2005). The blades that curved upward transversely and downward longitudinally in *rp-3* and *rp-5* leaves were similar to those of the *hyl1* mutant in which the function of HYL1 (responsible for miRNA biogenesis) is lost (Yu et al., 2005). The upward curling of *rp-5* was the same as that of *mir159ab* mutants (loss-of function mutants of *MIR159a* and *MIR159b* genes) (Allen et al., 2007). The *rp-7* leaves curved inward transversely. The leaflets in the *rc-5* blades were equivalent to those of plants overexpressing the miR164–targeted *CUC1* gene (Hasson et al., 2011).

To examine whether the leaf curvature of *B. rapa* was associated with miRNA accumulation, we chose the accessions *rc-1, rc-6, rp-2, rp-9*, and *rr-1* as representatives of the five types of leaf curvature (upward curving petioles, serrated blades, upward curving leaves, wavy margins, and downward curving leaves), respectively, for miRNA microarray analysis. There was great difference in the accumulation of miRNAs among the different genotypes (Table 2). Compared with *rp-2*, the accumulation of all types of miR169 in *rc-1, rc-6* and *rr-1* seedlings was increased by more than 2 times, while the accumulation of all types of miR172 and miR162 was decreased by 2 times. In *rp-9* seedlings, the accumulation of miR319ab was increased 2 times compared with *rc-6* plants, indicating that higher accumulation of miR319a is associated with the wavy leaf margins of *rp-9*. Compared with *rc-6* plants, which have flat leaves, miR166 accumulation in *rr-1* plants was increased by more than 1.5 times, suggesting that a relatively high level of miR166 is associated with the leaf downward curvature of the *rr-1* genotype.

**Table 2:** Changes in miRNA accumulation among genotypes of *B. rapa*.

**miRNAs**	***rc-1***	***rp-2***	***rc-6***	***rp-9***	***rr-1***
miR159c	~	↑	↑	↑	↑
miR162ab	↓	~	↓	↓	↓
miR164abc	↑	~	→	↓	→
miR166a-g	↑	→	~	←	↑
miR167ab	↑	→	~	→	↑
miR167c	↑	↑	~	→	↑
miR167d	↑	→	~	→	↑
miR168ab	↓	~	↓	↑	↓
miR169a	↑	~	↑	←	↑
miR169bc	↑	~	↑	←	↑
miR169defg	↑	~	↑	←	↑
miR169h-n	↑	~	↑	←	↑
miR172ab	↓	~	↓	→	↓
miR172cd	↓	~	↓	→	↓
miR172e	↓	~	↓	→	↓
miR319ab	↓	↓	~	↑	↓
miR319c	↓	↓	~	↑	↓
miR390ab	↑	↑	↑	↓	~
miR394a	↓	↓	~	↓	↓
miR395ade	↓	~	↓	←	↓
miR395bcf	↓	↓	~	←	↓
miR397a	↑	↑	~	↓	↑
miR398b	↑	↑	~	↑	↑
miR403	~	↑	↑	↑	↑
miR824	↑	↑	↑	↓	~
miR837a	↑	↑	~	↓	↑
miR848	↑	↑	~	↑	↑

*miRNA microarray analysis shows the relative accumulation of miRNAs among 5 genotypes. The symbol ~ indicates the reference. The symbols ↑ and ↓ indicate upregulation and downregulation, respectively, of more than 1.5-fold compared with the reference. The symbols → and ← Indicate an increase and a decrease, respectively, of less than 1.5-fold*.

### Association analysis of miR166 with types of leaf curvature

As mentioned above, the downward leaf curvature of some *B. rapa* accessions was comparable to that of the *jba-1D* mutant of *Arabidopsis*, while the wavy margins of some *B. rapa* accessions were similar to the *jaw-1D* mutant of *Arabidopsis*. We wondered whether high levels of miR166 were correlated with downward curvature in *B. rapa*. To address this question, we performed qPCR of miR166 using leaf samples of all 31 accessions showing downward curvature. Not all of the accessions with this type of leaf curvature showed higher levels of miR166. Among the accessions of the downward curving type in transverse direction, 84% showed upregulation of miR166 compared with *rp-9* (with upward curvature; Table 3). Similarly, among the accessions with wavy margins, 89% exhibited upregulation of miR319a compared with *rp-2* (without wavy margins). These results showed that high levels of miR319a were correlated with wavy margins.

**Table 3:** Association analysis of miRNA levels with the direction of leaf curvature.

**miRNAs**	**No. of accessions**		**Upregulation**		***r***
	**Downward**	**Wavy margin**	**No. of accessions (>1.5-fold)**	**%**	
miR166	31	~	26	84	0.76^*^
miR319a	~	18	16	89	0.69^*^

*q-PCR of miRNA expression was performed with three biological replicates. miR166 expression was normalized to that of rp-9 and miR319a expression to that of rp-2. The symbol ~ indicates no data are available. r indicates the correlation coefficient between miR166 expression (X) and curvature indices (Y) of the genotypes with downward curving leaves or between miR319a expression (X) and wave height (Y) of the genotypes with wavy margins*.

* *Indicates significant difference*.

### Overexpression of the MIR166g gene changes the direction and degree of leaf curvature

The genome of Chinese cabbage is mesohexaploid and thus most of the genes in Chinese cabbage have 2–3 copies, showing duplication or triplication. However, the *Brp-MIR165/6* gene is not in accordance with this rule. There are only 3 *Brp-MIR165* loci and 7 *Brp-MIR166* loci in Chinese cabbage, compared with 2 and 7 in *Arabidopsis*, respectively. By contrast, the miR165/6-targeted genes are duplicated or triplicated. This suggests that miR165/6-guided gene silencing in *B. rapa* is more complex than in *Arabidopsis*.

**Figure 7:**
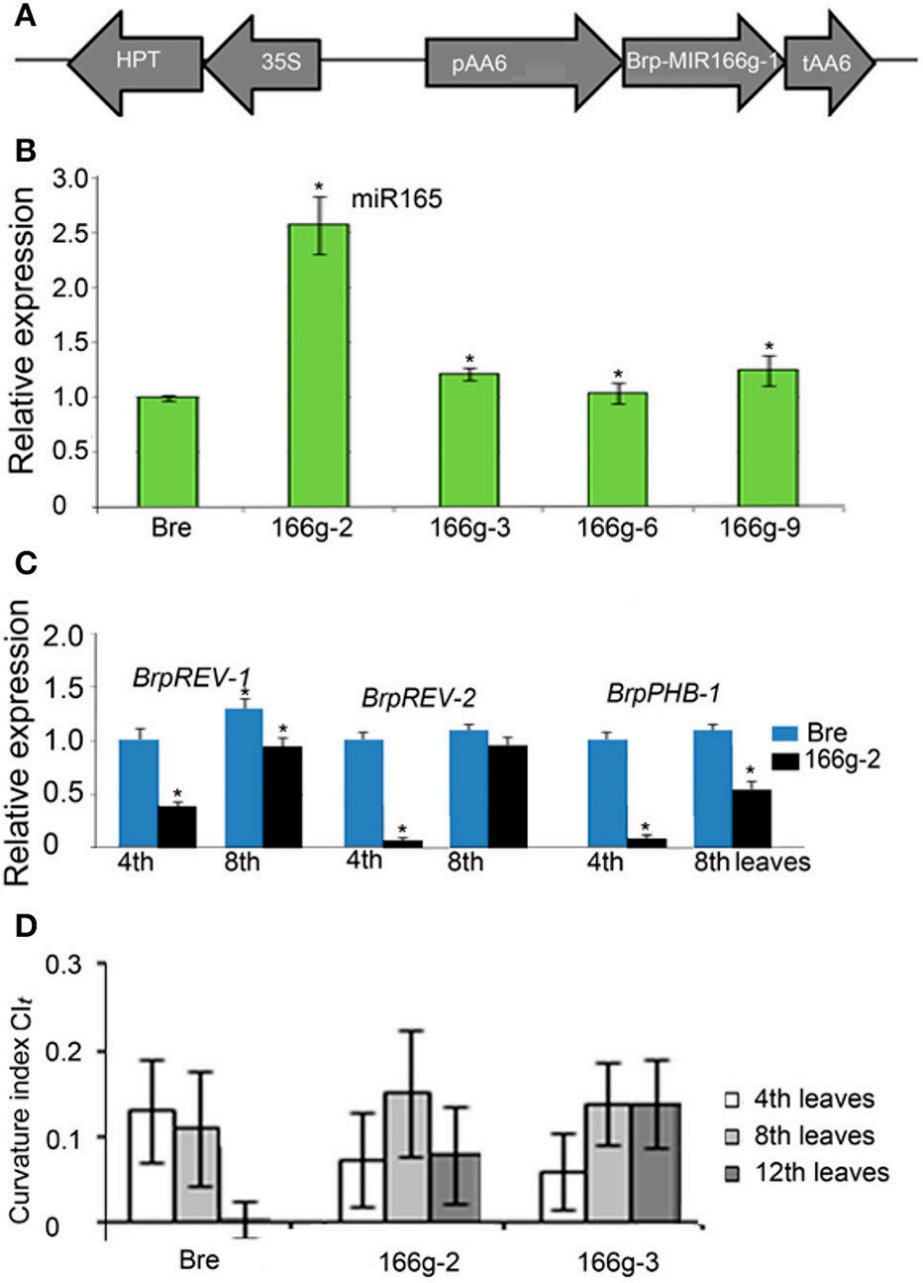
Expression levels of miR165 and its target genes, and curvature indices of leaves in transgenic lines of Bre (heading Chinese cabbage) carrying *pAA6::BrpMIR166g*. **(A)** Diagram of the *pAA6::BrpMIR166g* construct in the binary vector. **(B)** Expression levels of miR165 in the transgenic lines carrying *pAA6::BrpMIR166g*. **(C)** Expression levels of *BrpREV-1, BrpREV-2* and *BrpPHB-1* in the transgenic lines carrying *pAA6::BrpMIR166g*. **(D)** Transverse curvature indices of the leaves in the transgenic lines carrying *pAA6::BrpMIR166g*. The significant differences were referred to *P* < 0.001 (Student's *t*-test) between genotypes. ^*^Indicates the significant difference.

To verify the relationship between miRNA and leaf curvature in *B. rapa*, we constructed binary vectors of some *MIRNA* genes under the control of the AA6 promoter and overexpressed them in Chinese cabbage (Figure 7A). *Brp-MIR166g* is the most homologous to *MIR166g* within the *MIR165/6* gene family of *Arabidopsis*, but has a “T”-to-“C” substitution in the miR166 region and generates a mature miR165 rather than a miR166 (Supplemental Figure S1). *Brp-MIR166g* was placed under the control of the AA6 promoter and transferred into heading Chinese cabbage Bre. Through PCR of the AA6 promoter, nine transgenic lines (166g-1 through 166g-9) were identified (Supplemental Figure S2). Among them, the 166g-2 line showed a 2.5-fold increase in miR165 accumulation compared with the wild-type (Figure 7B). In the 4th leaves of this transgenic line, *BrpREV-1, BrpREV-2*, and *BrpPHB-1* were downregulated more than 2-fold (Figure 7C). This result indicated that overexpression of *Brp-MIR166g* caused the upregulation of miR165 and downregulation of its target genes.

**Figure 8:**
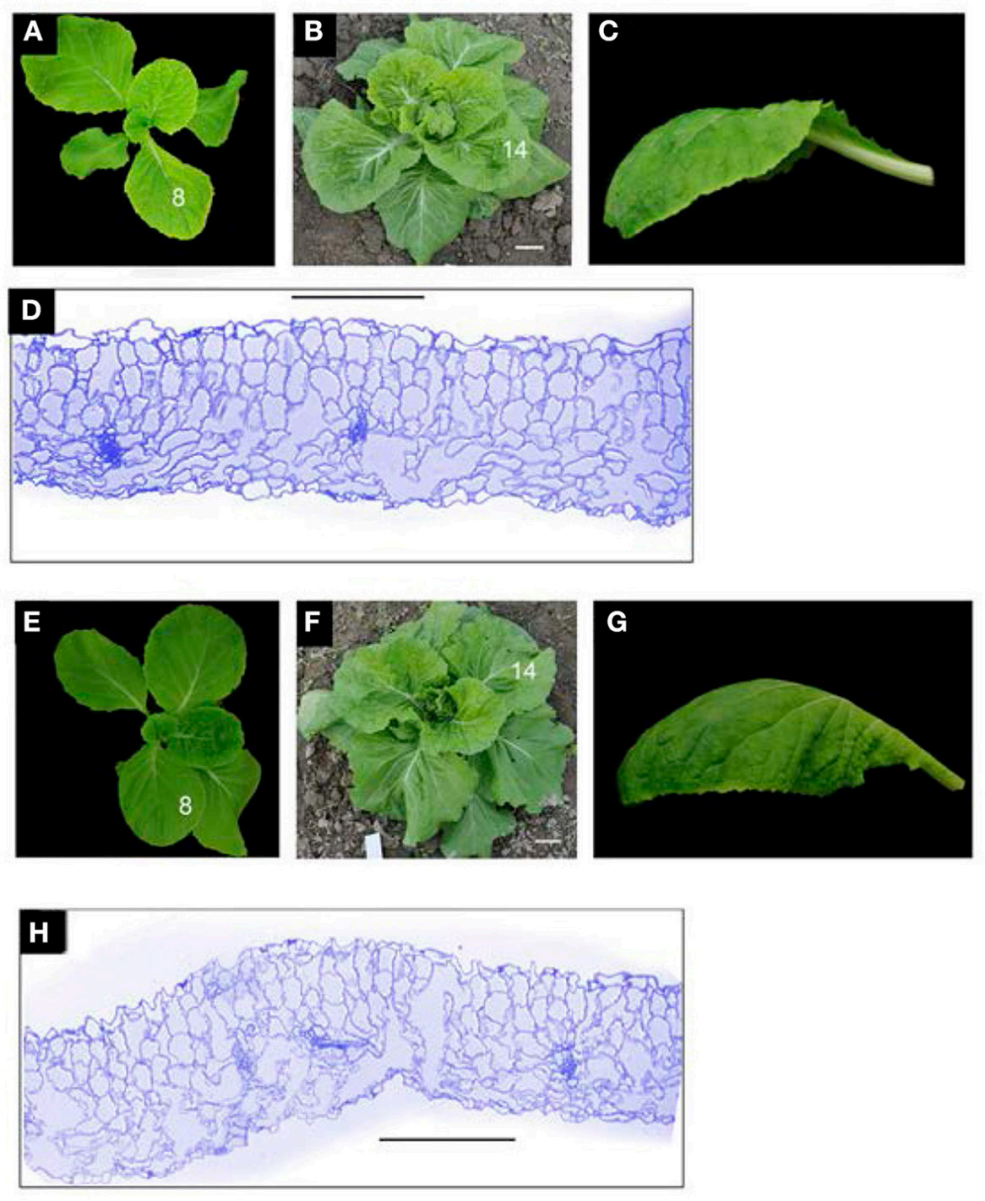
miR165/6 regulates the direction and degree of leaf curvature. **(A–C)** Seedling **(A)**, rosette **(B)** and 14th leaf **(C)** of the wild-type. **(D)** Transverse section of the 14th leaf of the wild-type showing adaxial/abaxial polarity. **(E–G)** Seedling **(E)**, rosette **(F)** and 14th leaf **(G)** of the 166g-2 line. **(H)** Transverse section of 14th leaf of 166g-2 showing the defects in adaxial/abaxial polarity. Bar = 200 μm.

In the field, the seedling leaves (8th leaves) of wild-type plants and 166g-2 plants carrying pAA6::*Brp-MIR166g* were downwardly curved (Figures 7D, 8A,D), and the rosette leaves (12th leaves) of the wild-type were basically flat (Figures 8B,C) whereas the rosette leaves of 166g-2 and 166g-3 plants were downwardly curved (Figures 7D, 8F,G). This demonstrated that higher accumulation of miR165 changed the direction and degree of leaf curvature at the rosette stage. When the young leaves on the shoot tip of the wild-type were upwardly and inwardly curved, those of 166g-2 plants were downwardly curved (Figures 8B,F). Because of the delay of leaf incurvature, the head size of 166g-2 plants was decreased and the heading time was delayed.

We noticed that the transverse CI of the 12th leaves in 166g-3 plants was higher than that of the 166g-2 plants (Figure 7D). Considering that the expression level of miR165 in 166g-3 plants was lower than in 166g-2 plants, this suggested that the expression levels of miR165 affected the degree of downward curvature of the rosette leaves in the transgenic plants carrying *pAA6::Brp-MIR166g*.

Overexpression of the *Brp-MIR166g* gene altered the adaxial–abaxial polarity of the rosette leaves. Compared with the wild-type, the adaxial regions in 166g-2 leaves contained many long cells similar to those in the abaxial regions of the wild-type (Figures 8D,H), and the intercellular spaces between the palisade mesophyll cells were larger than those of the spongy mesophyll cells of the wild-type. This result suggested that the adaxial regions in 166g-2 leaves had abaxial characteristics.

## Discussion

The formation of a normal leaf goes through stages of leaf identity specification, leaf polarity establishment, cell division and expansion control, and vascular pattern formation. Three-dimensional imaging has enabled quantitative characterization of the direction of the surface and the shape of leaves (Weight et al., 2008). According to the direction, axis and position of curvature, we classified 56 accessions of *B. rapa* into five leaf curvature types. However, leaf curvature in *B. rapa* is complex and could be sub-classified further when the global and local curvatures of leaves were examined more precisely. In many cases, leaf curvature was intertwined with the leaf incline angle, giving rise to more diverse phenotypes.

*B. rapa* and *A. thaliana* belong to the Crucifer family and share similar sequences in many genes. Many types of leaf curvature in *B. rapa* are similar to those reported in *Arabidopsis* mutants deficient in miRNA pathways. The wavy leaf margins of *rp-6* are concurrent with the upregulation of mi319a and look like those of *jaw-1D* mutants of *Arabidopsis* in which the *MIR319a* gene is activated (Palatnik et al., 2003). The apical upwardly curved region in *rc-5* is the same as in *dcl1-9* leaves (Liu et al., 2011). The lateral downwardly curved region in *rr-1* is consistent with the upregulation of miR166 and resembles that of the *jba-1D* mutant in which *MIR166g* is activated (Williams et al., 2005). The serrated leaf margins of *rp-6* are concurrent with the upregulation of mi164 and look similar to the *mir164a-4* mutant in which MIR164 is mutated (Nikovics et al., 2006). It is not known whether high accumulation of miR169 and/or low accumulation of miR162 contributes to leaf upward curvature. However, the possibility that these miRNAs affect leaf curvature cannot be excluded.

miRNA expression levels are associated with different types of leaf curvature. qPCR of miRNAs revealed that a higher level of miR166 was associated with downward curvature while higher miR319a expression was correlated with wavy margins. The phenotyping of different transgenic lines indicated that the expression levels of miR165 affect the degree of downward curvature of the rosette leaves in transgenic plants carrying *pAA6::Brp-MIR166g*. The inference is that variation of leaf curvature in *B. rapa* is related to some miRNAs and their target genes during evolution. Thus, it is important to study the molecular mechanism underlying the regulation of leaf curvature by miRNAs.

Heading Chinese cabbage goes through four developmental stages during its vegetative growth. The leaves at the seedling, rosette, folding, and heading stages are downwardly curved, flat, upwardly curved and inwardly curved, respectively. Overexpression of *Brp-MIR166g* genes caused a range of morphological changes with regard to leaf curvature including downward curvature of the rosette leaves and flatness of the folding leaves. The reasons for the changes in the direction and degree of leaf curvature are that *BrpREV-1, BrpREV-2* and *BrpPHB-1* were downregulated more than 2-fold.

Many miRNAs contribute to leaf curvature. Previous studies have shown that miR156 controls the timing of leaf curvature (Wang et al., 2014) and miR319a regulates cell division in the leaf tip (Mao et al., 2014). In the present study, we found that miR166 affected the type of leaf curvature. Apparently, the precise regulation of miRNAs is important for the morphological control of leaf curvature. Any mutation of the genetic elements upstream and downstream of these miRNAs may cause morphological changes in leaf curvature. We wonder whether other miRNAs regulate leaf curvature through their target genes. In fact, the levels and activities of other miRNAs such as miR164, miR169, and miR396 varied with the type of leaf curvature. For this reason, the characterization of different types of leaf curvature is important for exploring the relationship between individual miRNAs and leaf curvature.

## Author contributions

WR and HW performed experiments and drafted the manuscript; JB did genetic transformation; FW performed phenotyping; YH designed the research plan and wrote the manuscript.

### Conflict of interest statement

The authors declare that the research was conducted in the absence of any commercial or financial relationships that could be construed as a potential conflict of interest.
