# Supplementary figures and images for: Association of microRNAs with Types of Leaf Curvature in Brassica rapa
Source: Front Plant Sci. 2018 Feb 6;9:73. doi: 10.3389/fpls.2018.00073 (PMC5808167; doi:10.3389/fpls.2018.00073)

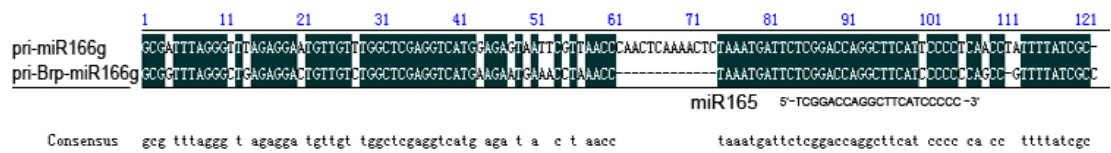

**Supplemental Fig. S1:** Alignment of pri-Brp-miR166g in *B. rapa* with pri-miR166g in *Arabidopsis*.

Supplement: Supplementary file 2 [file Image1.pdf]
