# Supplementary material for: Association of microRNAs with Types of Leaf Curvature in Brassica rapa
Source: Front Plant Sci. 2018 Feb 6;9:73. doi: 10.3389/fpls.2018.00073 (PMC5808167; doi:10.3389/fpls.2018.00073)
Supplement: Supplementary file 3 [file Image2.pdf]

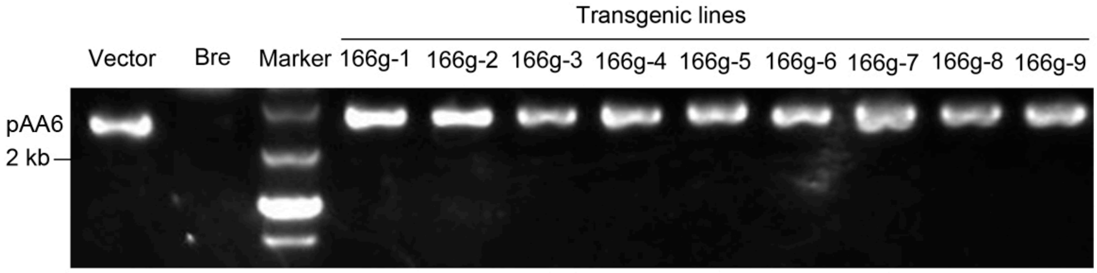


**Supplemental Fig. S2:** PCR detection of AA6 promoter in the transgenic lines of heading Chinese cabbage carrying *pAA6::Brp-MIR166g*.
